# New Ther1-derived SINE Squam3 in scaled reptiles

**DOI:** 10.1186/s13100-021-00238-y

**Published:** 2021-03-22

**Authors:** Nikita S. Vassetzky, Sergei A. Kosushkin, Vitaly I. Korchagin, Alexey P. Ryskov

**Affiliations:** 1 Institute of Gene Biology, Russian Academy of Sciences, Moscow, 119334 Russia; 2 Engelhardt Institute of Molecular Biology, Russian Academy of Sciences, Moscow, 119991 Russia

**Keywords:** SINEs, Retrotransposons, Squamata, Reptilia, Evolution

## Abstract

**Background:**

SINEs comprise a significant part of animal genomes and are used to study the evolution of diverse taxa. Despite significant advances in SINE studies in vertebrates and higher eukaryotes in general, their own evolution is poorly understood.

**Results:**

We have discovered and described in detail a new Squam3 SINE specific for scaled reptiles (Squamata). The subfamilies of this SINE demonstrate different distribution in the genomes of squamates, which together with the data on similar SINEs in the tuatara allowed us to propose a scenario of their evolution in the context of reptilian evolution.

**Conclusions:**

Ancestral SINEs preserved in small numbers in most genomes can give rise to taxa-specific SINE families. Analysis of this aspect of SINEs can shed light on the history and mechanisms of SINE variation in reptilian genomes.

**Supplementary Information:**

The online version contains supplementary material available at 10.1186/s13100-021-00238-y.

## Background

Genomes are invaded by various repetitive elements, the most abundant of which (at least in higher eukaryotes) are Long and Short INterspersed Elements (LINEs and SINEs, respectively). The amplification cycle of these retrotransposons includes the transcription of their genomic copies, reverse transcription and integration into the genome. LINEs rely on the transcription by the cellular RNA polymerase II, while reverse transcription and integration are fulfilled by their own enzymes. SINEs do not encode any enzymes and employ the cell machinery for their transcription by RNA polymerase III (pol III) and the machinery of their partner LINE for their reverse transcription and integration into chromosomes. Accordingly, SINEs have pol III promoters for transcription and sequences recognized by the enzymes of their partner LINE for reverse transcription/integration.

A typical SINE consists of the head derived from one of the cellular RNA species (tRNA, 7SL RNA, or 5S RNA); the body, the terminal part of which is recognized by the partner reverse transcriptase (RT); and the tail, a stretch of simple repeats. There are variations; certain SINEs have no body or their body contains sequences of unknown origin and function (some of them called central domains) that are shared between otherwise unrelated SINE families, etc. [1].

LINEs are found in the genomes of all higher eukaryotes. Clearly, SINEs cannot exist without LINEs but not vice versa; there are rare genomes that have LINEs but lack SINEs (e.g., *Saccharomyces* or *Drosophila*). During evolution, LINE (sub)families can become inactive and their partner SINEs also cease to amplify. If another LINE family becomes active in a particular genome, replacement of the sequence recognized by its RT can reanimate a SINE [2]. Usually, a genome harbors one or several SINE families; some of them can be inactive and were amplified in the ancestors. The analysis of SINE variation in different taxa allows us to use them as reliable phylogenetic markers [3, 4].

The main lineages of the reptile-bird clade are scaled reptiles (Squamata), tuatara (Rhynchocephalia), turtles (Testudines), crocodiles (Crocodilia), and birds (Aves). Squamata, the largest order of reptiles, include the following major lineages: Serpentes (snakes), Iguania (including iguanids, agamids, chameleons), Anguimorpha, Scincomorpha, Lacertoidea, Gekkota, and Amphisbaenia. Phylogenetic relations among squamate reptiles are highly controversial due to the conflicting signals provided by molecular, morphological, and paleontological data. Together with tuatara, the only extant representative species of Rhynchocephalia, they form monophyletic superorder Lepidosauria, which is the sister group to Archelosauria, the clade that contains archosaurs (crocodiles and birds) and turtles [5].

The first reptile SINE was found in 1990 in the Chinese pond turtle [6]; currently, we know approximately ten SINE families in reptiles [1] with a different taxonomic distribution, e.g., Cry is limited to turtles and degraded copies of AmnSINE, which was active in the ancestor of amniotes [7], can be found far beyond reptiles. Another example is Ther1 initially described as a mammalian SINE (MIR) but renamed later [8, 9]. Several known Ther1/MIR subfamilies (MIRb, MIRc, and MIR_Testu) have minor differences from Ther1 except the *Alligator mississippiensis’s* MIR1_AMi with an extended deletion. Moreover, active Ther1/MIR SINEs were found in non-avian reptiles, so ample and diverse derived SINEs could be expected in their genomes [10]. This is further corroborated by active diversification of reptilian L2 [11].

Despite active sequencing of genomes of various species of lizards and snakes, no detailed comparative genomic studies of a SINE family in different taxa at the order level are available. We discovered a new SINE named Squam3 in the genomes of *Darevskia* and *Anolis* lizards. Further analysis demonstrated their distribution throughout squamates; a similar SINE was found in the tuatara [12] but not in other reptiles or birds. However, Squam3 remained unnoticed in almost 40 genomes of squamates. Here, we analyzed the structure, distribution, and evolution of Squam3 and its relatives.

## Results

### Squam3 identification

The consensus sequence of *Darevskia* Squam3 was used to search the genomes of scaled reptiles. It was found in all sequenced genomes (as well as in a variety of GenBank sequences of squamate species whose genomes have not been sequenced; Table S1). No Squam3 was found beyond Squamata (see below). The analysis of their consensus sequences has revealed three major subfamilies that we called Squam3A, Squam3B, and Squam3C.

### Squam3 structure

Squam3 is a typical SINE [13] composed of the tRNA-derived *head*, the *body* with a central domain and the 3′-terminus matching that of the partner LINE, and the *tail*, a stretch of several simple repeats. The consensus sequences range from 218 to 239 nt (without tail). There is no clear preference for a particular tRNA species (which is not uncommon among SINEs).

**Fig. 1:**
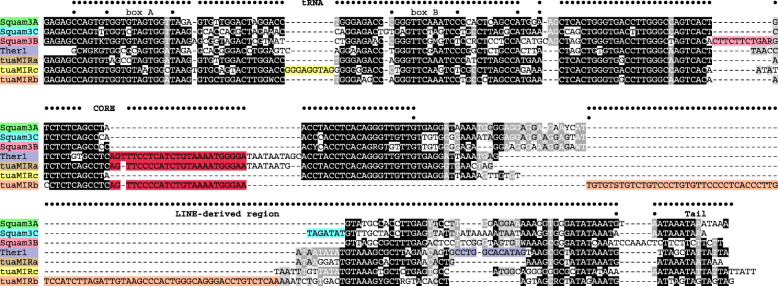
Sequence alignment of Squam3 subfamilies of squamate reptiles with tuatara tuaMIR SINEs and Ther1. The tRNA-derived region, CORE central domain, LINE-derived region, and tail are indicated above the sequences. See text for other explanations

The *body* is similar to a fragment of the CORE central domain; the pronounced similarity spans over 28 nt (double-overlined in Fig. 1). There is also a similarity with the very 3′-terminus of LINEs of the L2 clade identified in *Darevskia valentini* (data not shown) and a less pronounced similarity with L2 LINEs of *Anolis carolinensis* (L2-26_ACar and L2-24_ACar in Repbase).

The tail of Squam3 is largely composed of (TAAA)_n_ or (CTT)_n_; however, certain species have (GTT)_n_, (ATT)_n_, or poly(A) (Table 1). Squam3 has a very low rate of target site duplications. This is unusual but not exceptional among SINEs and can point to an alternative cleavage pattern in different DNA strands by the partner LINE endonuclease [13].

**Table 1:** Squam3 SINE in scaled reptiles. Major subfamilies are described by the proportion and estimated number of full-length copies, the mean sequence similarity, and the tail repeat unit. Certain parameters of genome assemblies are given in the left columns (the level column indicates the chromosome-, scaffold-, and contig-levels levels of assembly: ◕, ◑, and ◔, respectively)

		Family	Species	Squam3 SINE					Genome assembly			
				subfamily	copies	lengthw/o tail, nt	similarity	tail	level	‘N’	N_**50**_	Reference
**Squamata**	Gekkota	Gekkonidae	*Gekko japonicus*	3A (21%)	54,829	224	60%	(TAAA)_n_	◑	4%	707,733	[14]
				3B (10%)	26,109	238	75%	(CTT)_n_				
				3B3 (69%)	180,151	271	81%	(CTT)_n_				
			*Paroedura picta*	3A (12%)	17,761	221	57%	(TAAA)_n_	◑	9%	4,106,116	[15]
				3B (19%)	28,122	238	61%	(CTT)_n_				
				3B3 (57%)	84,367	267	74%	(CTT)_n_				
		Eublepharidae	*Eublepharis macularius*	3A (50%)	68,299	224	60%	(TAAA)_n_	◔	2%	663,762	[16]
				3B (50%)	63,045	239	85%	(GTT)_n_				
	Lacertoidea	Lacertidae	*Darevskia valentini*	3A (48%)	25,848	218	63%	(TAAA)_n_	◔	16%	658,539	[17]
				3B (52%)	28,003	238	91%	(CTT)_n_				
			*Lacerta agilis*	3A (16%)	17,446	219	62%	(TAAA)_n_	◕	0%	86,565,987	[18]
				3B (84%)	91,590	238	92%	(CTT)_n_				
			*Lacerta bilineata*	3A (39%)	24,123	219	63%	(TAAA)_n_	◔	0%	368,212	[19]
				3B (61%)	37,732	238	75%	(CTT)_n_				
			*Lacerta viridis*	3A (39%)	24,836	219	61%	(TAAA)_n_	◔	0%	662,519	
				3B (61%)	38,847	238	94%	(CTT)_n_				
			*Podarcis muralis*	3A (35%)	16,967	220	61%	(TAAA)_n_	◕	0%	92,398,148	[20]
				3B (65%)	31,440	238	88%	(CTT)_n_				
			*Zootica vivipara*	3A (38%)	10,036	220	61%	(TAAA)_n_	◕	3%	92,810,032	[21]
				3B (62%)	16,374	238	89%	(CTY)_n_				
		Teiidae	*Salvator merianae*	3A (53%)	4892	221	54%	(TAAA)_n_	◑	2%	55,382,274	[22]
				3B (47%)	4338	234	85%	(CTT)_n_				
	Serpentes	Colubridae	*Pantherophis guttatus*	3C	12,936	226	64%	(TAAA)_n_	◑	5%	16,790,024	[23]
			*Pantherophis obsoletus*	3C	12,961	226	63%	(TAAA)_n_	◑	3%	14,519,768	[24]
			*Ptyas mucosa*	3C	19,524	226	63%	(TAAA)_n_	◑	3%	15,963,960	[25]
			*Thamnophis elegans*	3C	16,934	226	64%	(TAAA)_n_	◕	0%	440,193	[26]
			*Thamnophis sirtalis*	3C	12,410	226	63%	(TAAA)_n_	◑	21%	647,592	[27]
			*Thermophis baileyi*	3C	15,914	228	65%	(TAAA)_n_	◑	8%	2,413,955	[28]
		Elapidae	*Emydocephalus ijimae*	3C	15,782	226	62%	(TAAA)_n_	◑	0%	18,937	[29]
			*Hydrophis cyanocinctus*	3C	15,094	226	64%	(TAAA)_n_	◑	4%	7437	[30]
			*Hydrophis hardwickii*	3C	15,782	228	62%	(TAAA)_n_	◑	4%	5391	[31]
			*Hydrophis melanocephalus*	3C	14,271	226	63%	(TAAA)_n_	◑	11%	59,810	[29]
			*Laticauda colubrina*	3C	19,118	226	63%	(TAAA)_n_	◑	13%	3,139,541	
			*Laticauda laticaudata*	3C	27,835	226	61%	(TAAA)_n_	◑	0%	39,330	
			*Naja naja*	3C	10,813	226	64%	(TAAA)_n_	◕	6%	224,088,900	[32]
			*Notechis scutatus*	3C	27,122	226	63%	(TAAA)_n_	◑	5%	5,997,050	[33]
			*Ophiophagus hannah*	3C	11,613	226	63%	(TAAA)_n_	◑	13%	241,519	[34]
			*Pseudonaja textilis*	3C	17,187	226	65%	(TAAA)_n_	◑	2%	14,685,528	[35]
		Pythonidae	*Python bivittatus*	3A (43%)	9349	221	58%	(TAAA)_n_	◑	4%	213,970	[36]
				3C (57%)	12,393	237	75%	(A)_n_				
		Viperidae	*Crotalus horridus*	3C	15,006	226	63%	(TAAA)_n_	◑	12%	23,829	[37]
			*Crotalus pyrrhus*	3C	15,556	226	64%	(TAAA)_n_	◑	0%	5299	[38]
			*Crotalus viridis viridis*	3C	18,694	226	63%	(TAAA)_n_	◕	6%	179,897,795	[39]
			*Protobothrops flavoviridis*	3C	20,667	226	64%	(TAAA)_n_	◑	3%	467,050	[40]
			*Protobothrops mucrosquamatus*	3C	20,184	228	64%	(TAAA)_n_	◑	8%	424,052	[41]
			*Vipera berus berus*	3C	19,964	226	64%	(TAAA)_n_	◑	14%	126,452	[42]
	Shinisauria	Shinisauridae	*Shinisaurus crocodilurus*	3A	165,288	225	58%	(TAAA)_n_	◑	8%	1,469,749	[43]
	Anguimorpha	Anguidae	*Dopasia gracilis*	3A	35,118	225	71%	(TAAA)_n_	◑	3%	1,273,270	[44]
	Varanoidea	Varanidae	*Varanus komodoensis*	3A	108,651	229	66%	(TAAA)_n_	◑	1%	23,831,982	[45]
	Iguania	Agamidae	*Pogona vitticeps*	3A	4542	221	53%	(TAAA)_n_	◑	4%	2,290,546	[46]
		Dactyloidae	*Anolis carolinensis*	3A	457	217	54%	(TAA)_n_	◕	5%	150,641,573	[47]
**Rhynchocephalia**			*Sphenodon punctatus*	─	─	─	─	─	◑	10%	3,052,611	[12]
**Testudines**			*Trachemys scripta elegans*	─	─	─	─	─	◕	1%	147,425,149	[48]
**Crocodilia**			*Crocodylus porosus*	─	─	─	─	─	◑	5%	84,437,661	[49]
**Aves**			*Gallus gallus*	─	─	─	─	─	◕	1%	91,315,245	[50]

### Squam3 subfamilies

Genomic copies of SINEs are subject to random mutations; accordingly, single-nucleotide mutations can be used to identify subfamilies only for highly conserved SINEs. We use extended insertions/deletions to distinguish between the three major Squam3 subfamilies designated as Squam3A, Squam3B, and Squam3C (Fig. 1). Squam3B has a characteristic 11-nt insertion (marked in pink in Fig. 1), and Squam3C has a characteristic 7-nt insertion (marked in blue in Fig. 1). There are also minor differences between the Squam3 subfamilies. In addition, there are sub-subfamilies; one of these (Squam3B3) has become a major variant in the two Gekkonidae species.

Further analysis of Squam3-related sequences in the tuatara genome has revealed a similar SINE (tuaMIRa) with a 32-nt insertion (marked in amaranth in Fig. 1). This insertion restores the CORE central domain and makes the element similar to Ther1 (MIR). It should be noted that this deletion in Squam3 and tuaMIRс relative to Ther1 is distinct from the deletion in MIR1_AMi (Fig. S2A). TuaMIR SINEs also have an 8–13-nt deletion in the LINE-derived region (marked in violet in Fig. 1). Moreover, another element (tuaMIRb) with a similar insertion lacks the ~ 40-nt region between the CORE and the LINE-derived region conserved in other Squam3- and Ther1-related SINEs but has a much longer L2 LINE-derived region due to the 77-nt insertion (marked in mango in Fig. 1). The sequences of these tuatara SINE families were recently reported [12] but only the relation to MIR (former name of Ther1) and the mean divergence of all Ther1-related sequences were mentioned.

**Fig. 2:**
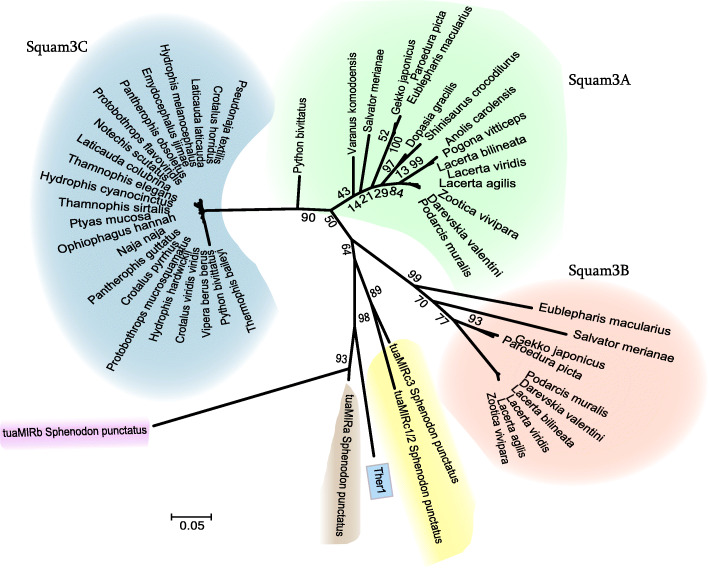
Unrooted NJ tree of consensus sequences of Squam3 and tuaMIR SINEs

Apart from that, Squam3 subfamilies differ by the tail, which is largely (TAAA)_n_ in Squam3A/C or (CTT)_n_ in Squam3B. The mean sequence similarity also differs between subfamilies, it peaks in Squam3B (up to 94%) but is lower in Squam3C (~ 63%) and Squam3A (54–63%). Figure 2 visualizes the diversity of Squam3 in the genomes of lizards, snakes, and tuatara. Squam3C in most snake species demonstrates little variation between species; this contrasts with the diversity within Squam3A and Squam3B subfamilies. The tuatara SINEs clearly constitute a cluster separate from Ther1.

**Fig. 3:**
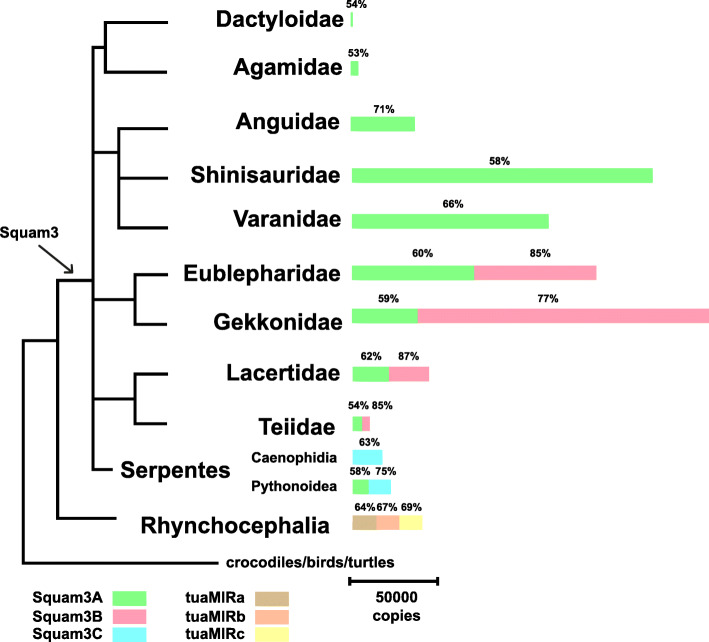
Schematic distribution of Squam3 SINEs in Squamata lineages studied. Colored band lengths are proportional to their copy numbers in genomes and the numbers above indicate the mean similarity of individual copies. If more than one species was available, the mean values are given

The number of Squam3 full-length copies varied over a wide range: from ~ 500 in *Anolis carolinensis* to ~ 260,000 in *Gekko japonicas* (0.005 and 2.55% of the genomes by length, respectively) (Fig. 3). The mean similarity of Squam3 subfamilies in most species is 60–65% with the notable exceptions of Squam3B (~ 90%) and Squam3A in Iguania (53%).

### Distribution of Squam3 in reptile genomes

We next searched for the consensus sequences of Squam3 subfamilies in genomes of squamates and neighboring taxa. Overall, the genomes of 38 squamates, tuatara, turtle (*Trachemys scripta elegans*), crocodile (*Crocodylus porosus*), and bird (*Gallus gallus*) were analyzed. Squam3 was found in all squamates but neither in other reptiles nor in birds (Table 1). Similar SINE families were found in the tuatara (*Sphenodon punctatus*). When this work was in progress, Gemmel et al. [12] reported these SINEs, so we use their nomenclature of tuatara SINEs.

The genomes of Gekkota and Lacertoidea (Gekkonidae, Eublepharidae, Lacertidae, and Teiidae families) had both Squam3A and Squam3B subfamilies in similar proportions (although the proportion of Squam3A could be occasionally as low as 12%). Snakes had the Squam3C subfamily except for the python, which had 43% Squam3A. The rest of the squamates (Shinisauridae, Anguidae, Varanidae, Agamidae, and Dactyloidae families) had the Squam3A subfamily alone (Table 1). The analysis of individual NCBI sequences of squamate species not listed in Table 1 largely confirms this pattern except that a few highly divergent Squam3A sequences were found in three more snake families (Elapidae, Lamprophiidae, and Viperidae) (Table S1). We specifically searched for Squam3A in one of the advanced snakes (*Vipera berus*), and found ~ 330 copies.

The tuatara (Sphenodontidae) has a set of tuaMIR families related to Squam3 and Ther1. Thus, we specifically searched for these sequences in the genomes of Squamata. No tuaMIRb or tuaMIRc were found, while minor tuaMIRa quantities exist in all squamate genomes analyzed ranging from a single full-length copy to ~ 500 (in *Shinisaurus crocodilurus*) (Table S2). All snakes have a single tuaMIRa copy in the same genomic locus (as judged by very similar flanking regions).

### Squam3 and other similar CORE SINEs

We compared Squam3 with tuaMIR and other CORE-containing SINEs of vertebrates. While the 5′-sequences of all COREs are similar, the characteristic deletion (marked in amaranth in Fig. 1) distinguishes all Squam3 and tuaMIRc from other SINEs (Fig. S2C).

## Discussion

One of the most intriguing aspects of SINEs is how they emerged and evolved. This study gives us a unique opportunity to trace this for a single SINE family in a very wide range of taxa. The Squam3 SINE was found in scaled reptiles (Squamata) but not in the tuatara (Rhynchocephalia) and further lineages including crocodiles, birds, and turtles. We found three major subfamilies distinguished by relatively long insertions/deletions (Squam3A, Squam3B, and Squam3C). They also differ by the number of copies and the mean sequence similarity, which points to the age of a SINE subfamily (to be precise, to the time of its amplification) since SINE genomic copies are not subject to selective pressure and gradually accumulate mutations with time.

### Evolution of Squam3

Overall, presumably there was a small pool (a few hundred?) of not very active Squam3A in the genomes of ancestral Squamata. In some lineages (Shinisauridae and Varanidae), Squam3A amplified quite actively without significant sequence modifications (to reach ~ 165,000 copies in *Shinisaurus crocodilurus*; the number of Squam3 copies was higher only in the *Gekko japonicus* with a ~ twice larger genome). Squam3A amplification was also active in Anguidae (~ 35,000 copies in *Dopasia gracilis*) but it started relatively recently considering the high mean similarity (71%) of the SINE sequences in this legless lizard. On the contrary, Squam3A gradually declined in Agamidae (~ 4500 copies and 53% mean similarity in *Pogona vitticeps*). Finally, Squam3A ceased to propagate (and evolve) in Dactyloidae (< 500 copies in *Anolis carolinensis*).

While other Squam3 subfamilies emerged in squamate lineages, Squam3A continued to amplify in Gekkota and Lacertoidea (from ~ 5000 to ~ 65,000 copies) but not in snakes (except primitive ones, ~ 9000 in *Python bivittatus*). We could find only ~ 300 copies in *Vipera berus*; individual copies were also found in non-genomic sequences of four other snake families (Table S2).

After Squam3A declined in the Gekkota and Lacertoidea, their genomes gave rise to the Squam3B subfamily. It is arguably the youngest Squam3 subfamily. Amazingly, the mean similarity of Squam3B is very high in *Lacerta agilis* (92%) and *L. viridis* (94%) but as low as 75% in *L. bilineata*. This indicates that Squam3B is likely active in *L. viridis* and *L. agilis* but not in *L. bilineata* representing the same genus. In Gekkonidae, the more prolific Squam3B3 sub-subfamily emerged (~ 180,000 copies in *Gekko japonicus*, which is the highest number of all Squam3 subfamilies). For some reason, the activity of both Squam3A and Squam3B was low in Teiidae (*Salvator merianae*) but still, Squam3B amplified later than Squam3A.

The Squam3C subfamily is limited to snakes; moreover, it is the only major subfamily in most snakes. Squam3A quantities were probably present in all squamates but did not propagate in most snakes. Instead, the Squam3C in advanced snakes (Caenophidia) became active slightly later or in the same period of time (the mean Squam3C similarity is 61–65% vs. 51–71% in Squam3A). This pattern is not true for *Python bivittatus* representing more primitive snakes, where the amplification of Squam3A was followed by that of Squam3C (with the mean similarities of 58 and 75%, respectively).

### Origin of Squam3

We were very excited to find what is called the “missing link” of Sqaum3 evolution in the tuatara. The genome of *Sphenodon punctatus* has three SINE families that are similar to Squam3 in the leftmost ~ 120 nt except the 32-nt deletion in Squam3 relative to two of them (tuaMIRa and tuaMIRb). Thus, a large CORE fragment was deleted in two tuaMIR SINEs. Another tuatara SINE (tuaMIRс) has this deletion and is similar to Squam3 within this region (but differs in the head and LINE-derived regions). It is plausible that the ancestor of Ther1 that was active in the common ancestor of mammals, reptiles, birds, and even coelacanth [9, 51] acquired the 32-nt deletion within the CORE domain in the Lepidosauria ancestor and the same region is present in related SINEs (Figs. S2B and S2C). This precursor SINE gave rise to tuaMIRс in the tuatara and Squam3 in Squamata.

## Conclusions

We discovered a new SINE Squam3 found in all (38 to the time of analysis) sequenced genomes of scaled reptiles (Squamata). Despite the ever-increasing amount of genomic data for lizards and snakes, this quite prolific SINE was not reported previously. The evolutionary dynamics of SINE families and subfamilies is obscure and linked to the divergence of the genomes. This study is a step forward in understanding how SINEs emerge and decline. We identified and described Squam3 subfamilies and directly compared their structural traits and copy number across a variety of major squamate taxa in comparison with related tuatara SINE families. This study gives an insight into how SINE families emerge and evolve.

## Methods

Most genomic data were downloaded from NCBI Genomes (https://www.ncbi.nlm.nih.gov/genome) except *Anolis carolinensis*, *Podarcis muralis* (Ensembl, https://www.ensembl.org), *Dopasia gracilis*, *Shinisaurus crocodilurus* (diArk, https://www.diark.org/diark), and *Darevskia valentini* [17]. We used the genomic sequences of *Lacerta agilis* and *Thamnophis elegans* with permission from the Vertebrate Genomes Project. Individual sequences of squamate species not listed in Table 1 were also extracted from NCBI (https://www.ncbi.nlm.nih.gov/taxonomy/advanced). If no data on the genome size was available in publications or the Animal Genome Size Database [52], it was calculated as the mean of most close species.

We used custom Perl scripts based on the Smith-Waterman search to find genomic copies of SINEs with at least 65% identity and 90% length overlap with the consensus. After all Squam3 families were identified, the genome bank was successively depleted using their consensus sequences and all hits were combined for further analysis.

Multiple sequence alignments were generated using *MAFFT* [53] and edited by *GeneDoc* [54]. Subfamilies were identified manually and analyzed in a larger sample if necessary. We considered only ample subfamilies (≥1% of the total number of full-length copies). A search for tuaMIR SINEs in reptile/bird genomes was carried out by initial identification of all copies with at least 65% similarity to the consensus sequences followed by manual subsampling and realigning of candidate copies possibly containing specific mutations separating them from tuaMIRa sequences. The mean similarity was determined for 100 randomly selected sequences (or all available if less) using the *alistat* program (Eddy S., Cambridge, [55]). A neighbor-joining tree was constructed using MEGA software with 1000 bootstrap replications and the “partial deletion” option.

## Supplementary Information

**Additional file 1: Fig. S1.** Alignment of species-specific Squam3 sequences. Green, Squam3A; red, Squam3B; blue - Suam3C. Species designations are: Squam3EmA, *Eublepharis macularius*; Squam3GjA, *Gekko japonicus*; Squam3PpA, *Paroedura picta*; Squam3Vk, *Varanus komodoensis*; Squam3Ch, *Crotalus horridus*; Squam3Pt, *Pseudonaja textilis*; Squam3Cp, *Crotalus pyrrhus*; Squam3Pg, *Pantherophis guttatus*; Squam3Nn, *Naja naja*; Squam3Oh, *Ophiophagus hannah*; Squam3Pmc, *Protobothrops mucrosquamatus*; Squam3Ts, *Thamnophis sirtalis*; Squam3Hc, *Hydrophis cyanocinctus*; Squam3Te, *Thamnophis elegans*; Squam3Pf, *Protobothrops flavoviridis*; Squam3Po, *Pantherophis obsoletus*; Squam3Cv, *Crotalus viridis*; Squam3Hh, *Hydrophis hardwickii*; Squam3Tb, *Thermophis baileyi*; Squam3Vb, *Vipera berus*; Squam3Ej *Emydocephalus ijimae*; Squam3Hm, *Hydrophis melanocephalus*; Squam3Ll, *Laticauda laticaudata*; Squam3Lc, *Laticauda colubrina*; Squam3Ns, *Notechis scutatus*; Squam3Pr, *Protobothrops mucrosquamatus*; Squam3PbC, *Python bivittatus*; Squam3DvB, *Darevskia valentini*; Squam3LbB, *Lacerta bilineata*; Squam3LaB, *Lacerta agilis*; Squam3LvB, *Lacerta viridis*; Squam3PmB, *Podarcis muralis*; Squam3ZvB, *Zootica vivipara*; Squam3GjB, *Gekko japonicus*; Squam3PpB and Squam3PpB3, *Paroedura picta*; Squam3GjB3, *Gekko japonicus*; Squam3EmB, *Eublepharis macularius*; Squam3SmB, *Salvator merianae*.

**Additional file 2: Fig. S2. A.** Alignment of Ther1/MIR subfamilies. **B.** Comparison of full-length consensus sequences of Squam3, tuaMIR and other CORE SINEs with tRNA- and L2-derived regions. The corresponding regions are indicated above the sequences. **C.** CORE domains of CORE SINEs in vertebrates. The characteristic Squam3 deletion is marked in amaranth (as in Fig. 1).

**Additional file 3: Fig. S3.** Alignment of LINE-derived regions of tuaMIRb and Ther1 and 3′-terminal sequences of several L2 LINEs. The origin and total length is given in parentheses.

**Additional file 4: Table S1.** Squam3 copies found in individual NCBI sequences of squamate species not listed in Table 1.

**Additional file 5: Table S2.** Distribution of tuaMIR subfamilies in genomes of animals studied.

## Data Availability

The data generated are available in the manuscript supporting files. The banks of Squam3 SINEs, as well as multiple alignments of random sets of SINE sequences, are available for each species on request.
