# Supplementary material for: New Ther1-derived SINE Squam3 in scaled reptiles
Source: Mob DNA. 2021 Mar 22;12:10. doi: 10.1186/s13100-021-00238-y (PMC7983390; doi:10.1186/s13100-021-00238-y)
Supplement: Supplementary file 2 — Additional file 2: Fig. S2. A. Alignment of Ther1/MIR subfamilies. B. Comparison of full-length consensus sequences of Squam3, tuaMIR and other CORE SINEs with tRNA- and L2-derived regions. The corresponding regions are indicated above the sequences. C. CORE domains of CORE SINEs in vertebrates. The characteristic Squam3 deletion is marked in amaranth (as in Fig. 1). [file 13100_2021_238_MOESM2_ESM.docx]

**A**

Ther1/MIR GCWGKGTGGCGCAGTGGATAGAGCACGGGACCTGGAGTCAGGAAGACCTGGGTTCGAATCCCGGCTCTGCCACTTACTAGCTGTGTGACCTTGGGCAAGTCACTTAACCTCTCTGTGCCTCAGTTTCCTCATCTGTAAAATGGGGATAATAATAGC------ACCTACCTCACAGGGTTGTTGTGAGGATTAAATGAGA-TAATATATGT-AAAGCGCTTAGA----ACAGTGCCTGGCACATAGTAAGCGCTATATAAATGTTAGCTATTATTA -
MIRb CAGAGGGGCAGCGTGGTGCAGTGGAAAGAGCACGGGCTTTGGAGTCAGACAGACCTGGGTTCGAATCCCGGCTCTGCCACTTACTAGCTGTGTGACCTTGGGCAAGTTACTTAACCTCTCTGAGCCTCAGTTTCCTCATCTGTAAAATGGGGATAATAAT---------ACCTACCTCGCAGGGTTGTTGTGAGGATTAAATGAGA-TAATGCATGT-AAAGCGCTTAGC----ACAGTGCCTGGCACATAGTAAGCGCTCAATAAATGGTAGCTCTATTATT -
MIRc CGAGGCAGTGTGGTGCAGTGGAAAGAGCACTGGACTTGGAGTCAGGAAGACCTGGGTTCGAGTCCCGGCTCTGCCACTTACTAGCTGTGTGACCTTGGGCAAGTCACTTAACCTCTCTGAGCCTCAGTTTCCTCATCTGTAAAATGGGGATAATAATACCTGCCCTGCCTACCTCACAGGGTTGTTGTGAGGATCAAATGAGA-TAATGTATGTGAAAGCGCTTTGT----AAACTGTA-----------AAGCGCTATACAAATGTAAGGNGTTATTATTATT
MIR1_AMi TAGGGAGGCAGTGTGGTCTAGTGGATAGAGCACTGGACTGGGACTC-GGGAGACCTGGGTTCTATTCCCGGCTCTGCCACTGGCCTGCTGGGTGACCTTGGGCAAGTCACTTCACCTCTCTGTGCCTCAGTTTCCCCATCTGTAAAATGGGGATAATGAT---------ACTGACCTC---------------------------------CTTTGT-AAAGTGCTTTGA----GATCTACT-GATGAA----AAGTGCTACATAAGAGCTAGGTATTATTAT----
MIR_Testu CAGAGAGGAAGGATGGTCCAGTGGTTAGGGCGCTAGCCTGGGACTC-GGGAGACCCGGGTTCAATTCCCTGCTCCGCCACAGACTTCCTGTGTGACCTTGGGCAAGTCACTTAGCCTCTCTGTGCCTCAG-TTCCCCATCTGTAAAATGGGGATAATAGCACTT-----CCCTACCTCACAGGGGTGTTGTGAGGAT-AAAT-ACATTAAAGATTGT-GAGGCGCTCAGATACTACGGTGAT-----------GGGGGCCATATAAGTACCTAAGATAGATAGAT -

**B**

 ┌────────────────────────── ─────────────── **tRNA** ────────────────────────────────────┐┌─ ────────────────────────────── ──────────────── ─────────── CORE ────────────────────────────────────

│ ││
Mon1 AGAGAAGCAGCGTGGCCTAGTGGATAGA-GCACGGGCCTGGGAGTC--------AGAAGGACC-TGGGTTCTAATCCCGGCTCCGCCACTTG------TCTGCTGTGTGACCTTGGGCAAGTCACTTM-------ACTTCTCTGTGCCTCA-GTTACCTCATCTGTA-AAATGGGGATTAAGACTGTGAGCCCCACGTGGG----
Ther1 GCWGKGTGGCGCAGTGGATAGA-GCACGGGACCTGGAGTC--------AGGAAGACC-TGGGTTCGAATCCCGGCTCTGCCACTTA------CTAGCTGTGTGACCTTGGGCAAGTCACTTA-------ACCTCTCTGTGCCTCA-GTTTCCTCATCTGTA-AAATGGGGATAATAATAGC------------------
tuaMIRa GAGAGCCAGTGTAGCCTAGTGGATAAG-GTGTTGGACTTGGACC---------TGGGAGACC-AGGGTTCAAATCCCATCTTAGCCATGAA------CTCACTGGGTGGCCTTGGGCAAGTCACT-----------ATCTCTCAGCCTCA-GTT-CCCCATCTGTA-AAATGGGAATAATAATG--------------------
tuaMIRb GAGAGCCAGTGTGGTGTAGTGGATAAG-GTGCTGGACTTGGWCC---------TGGGAAGCC-AGGGTTCAAATCCTGCCCTAGCCATGAA------CTCACTGGGTGGCCTTGGGCAAGTCACA-----------ACCTCTCAGCCTCA-GTT-CCCCATCTGTA-AAATGGGAATG--------------------------
Coe2 GGGGGCAGTGTAGCGTAATGGTCAGA-ACACCTGACTGTGAAC---------CAGGAGACC-AGGGTTTGATTCCTGGCTCAGCCACTGA------CTCACTGTGTGACCTTGGGCAAGTCACTTG-------ACCTCCTTGTGCCTCA-GTTCCCCCAGCTGTA-AAATTGGGTGGAAGCAGGACCCCAC------------
Squam3A GAGAGCCAGTGTGGTGTAGTGGTTAGA-GTGTTGGACTAGGACC---------TGGGAGACC-CGGGTTCAAATCCCCACTCAGCCATGGAAG----CTCACTGGGTGACCTTGGGCCAGTCACT-----------GTCTCTCAGCCTA-------------------------------------------------------
Squam3C GAGAGCCAGTTTGGTCTAGTGGTTAAG-GCACCAGGCTAGAAAC---------CAGGAGACTGTGAGTTCTAGTCCTGCCTTAGGCATGAAAG----CCAGCTGGGTGACTTTGGGCCAGTCACT-----------CTCTCTCAGCCCA-------------------------------------------------------
tuaMIRc GAGAGCCAGTGTGGTGTAATGGCTAAGTGTGCAGTACTTGGACCGGGAGGTAGGGGGGGACC-CTGGTTCAAGTCTCGCCTTAGCCAGAAA------CTCACTGGGTGACCTTGGGCCAGTCACAAT--------ATTCTCTCAGCCTA-------------------------------------------------------
Squam3B GAGAGCCAGTKTGGTGTAGTGGTTAAGAGCGGTAGACTCGTAAT---------CTGGAGAAC-CGGGTTCGMGTCTCCRCTCCTCCACATGCAG---CTAGCTGGGTGACCTTGGGCTAGTCACACTTCTTCTGARGTCTCTCAGCCCC-------------------------------------------------------
Xt1 AGGAGT-TGGCCTAGAGGTTAAG-TGATCAGCCTTTGATG---------TGGGATCTC-ATGCTAGAGACCCTGGTTTGATTCCCTGTGGC--AACTCCTTGTGACCCTGGACAAGTCACTTA-------ATCTCCTGGTGTCCCAGCATACTTAGTG------------------------------------------
AFC GGGCGATCGTGGCTCAAGAGTTGG--GAGTTCGCCTTGTAAY---------CGGAAGGTTGCCGGTTCGAGCCCCGGCTCGGACAGTCT------CGGTCGTTGTGTCCTTGGGCAAGACACTTC-------ACCCGTTGCCTACTGGTGGTGGCCAGAGGGGCCGATGGCGCGATATGGCAGCCTCGCCTCTGTCAGTSYG
GA1 GGGCGACTGTGGGTGAGTGGGGAGC-ACGGTCGTCCTCCAAT---------CAGAGGGTTGTCGGTTCGATCCCAGGCCCGGCTAACCCGC----ATGTCGATGTGTCCTTGGGCAAGACACTTA-------ACCCAACATTGCTCCTGTAGCTGCGACTACAGTGTGTGAATGTTAGTTACTGATGGCAGGTGTCACTGTG
HpaI GGGGCGGCAGGGTAGCCTAGTGGTTAGA-GCGTTGGACTAGTAAC---------CGAAAGGTTGCAAGTTCAAATCCCCGAGCTGACAAGGTACAAATCTGTCGTTCTGCCCCTGAACAAGGCAGTTA-------ACCCACTGTTCCTAGGCCGTCATTGAAAATAAGAATTTGTTCTTAACTGACTTGCCTAGTTAAAT-----
Una1 CGGAGTGTAGCACAGTGGGTAAG-GAACTGGGCTTGTAAC---------CGAAAGGTCGCAGGTTCGATTCCCGGGTAGGACA----------CTGCCGTTGTACCCTTGAGCAAGGTACTTA-------ACCKGAATTGCTTCAGTATATATCCAGCTGTATAAATGGATACAATGTAAAATGCTATGTAAAAGTTG--
SP6 GTAGAGGTGTCGTGGTCGAGTGGATTAAGTCACTTGACTGTAGAT---------CACAAGGTCGAGGGTTCAARTCCCGCCACGGCAC----------------TAATGTCCTTTGGCAAGACA-TTA-------ATCTACATTTGCCACT-CTCCACCCAGGTGTT-AAATGGGTACCCGGTAGGATGCGAAAGTTAATGTGGT

 ────────────────────┐ ┌────────────────────── L2-derived region ───────────────────┐

│ │ │
Mon1 ------------------------------ACAGGGACTGTG------------------------------------------------------TCCAACCTGATTAGC-----------TTGTACTACCCCAGCGCTTAGAACAGTGCCTGGCACATA-GTAAGCGCTTAACAAATACCA-TAATTATTA
Ther1 ---------------------ACCTACCTCACAGGGTTGTTG--------------------------------------------------TGAGGATTAAATGAGATAAT--------------ATATGTAAAGCGCTTAGAACAGTGCCTGGCACATA-GTAAGCGCTATATAAATGTTAGCTATTATTA
tuaMIRa ---------------------ACCTACCTCACAGGGTTGTTG---------------------------------------------------TGAGGGTGAACTAGATAAG--------------GATTGTAAAGCACTTTGAACACT-------------GAAAGTGCTATATAAATGATAAATAATAAA
tuaMIRb ------------------------------------------TGTSTGTCTGTCCCTGTGTTCCCCTCACCCTTGTCCATCTTAGATTGTAAGCCCACTGGGCAGGGACCTGTCTCAAAAATCTGTGACTGTAAAGYGCTRGTACACCT-------------AGTAGCRCTATAGAAATGATTAGTAGTAGTAG
Coe2 -------------------------------------------------------------------------------------------------TGAAAATGAG---------------------ATGTCACATCTCAGTTGGGCTATCCTGGGTA---AACAATCTCATAAAA
Squam3A ---------------------ACCTACCTCACAGGGTTGTTG--------------------------------------------------TGAGGATAAAATGGGGAGGAGGAG--------AAYCATGTATGCCACCTTGAGYTCC-TTGGAGGAA---AAAGGTGGGATATAAATGTAATAAATAAATAAA
Squam3C ---------------------ACCCACCTCACAGGGTTGTTG--------------------------------------------------TTGTGGGGAAAATAGGAGGAGGAAGGAGTATTAGATATGTTTGCTACCTTGAGTTATTTATAAAAATAATAAAGGTGGGATATAAATAA-----ATAAATAAA
tuaMIRc ---------------------ACCTACCTCACAGGGTTGTTG--------------------------------------------------TGAGGATTAAAAGTTGTGTT------AATTTGTTATATGTAAAGTGCTCTGAGTGCC-ATGGCAGG---GGGGCGCTATATAAAAATAAATATTATTATTATT
Squam3B ---------------------ACYCACCTCACAGRGTGTTTG--------------------------------------------------TTGTGGGGAGAGGAAGGGAAAGGA----------GAWTGTTAGCCGCTTTGAGACTCCTTCGGGTAGTGRWAAAGCGGGATATCAAATCCAAACTCTTCTTCTTCTT
Xt1 ------------------------CACCTATAATGGCTGCCT--------------------------------------------------------------------------------TGCTTGCTGTAAAGCGCTTTGAGTCCC-ATGGG-----AGAAAAGCACTATATAAATAATTCCCTTTCCCTTTT
AFC CCCCAGGGYRGCTGTGGCTACAACTGTAGCTTGCCTYCAC----------------------------------------------------CAGTGTGTGAATGTGTGAGTGAAT----GAGTGGAATTGTAAAGCGCTTTGGGGTCC-TTAGGGACC-AGAAAAGCGCTATATAAATACAGTCCATTTAYYATTATTATTATT
GA1 TATGGTTCTCCTGTCATCAGTGTATGAA-----------------------------------------------------------------TGGGTGTGAATGGGTGAATGATG----TCATGTAGTGTTAAAGCGCTTTGAGTGGT-CAGAAGAC-TAGAAAAGCGCTATACAAGTACAGGCCATTTACCATTTA
HpaI ------------------------------------------------------------------------------------------------------------------------------------------------------------------AAAGGT
Una1 ---------------------------------------------------------------------------------------------------------------------------------TGTAAGTCGCTCTGGAT----------------AAGAGCGTCTGCTAAATGCCTGTAATGTAATGTAATG
SP6 TTGATTAGCAAGTGTGCGCTTGTAAAAATGACGCCTGGCTGGAATGCTCCCCAGGGAGTGGAGAATGTGCAYACATTGTGTGCGGG------TAAGGCCTGAATCCAATGACCGGGGTAATAATATATATGTAAAGCGCTTAGAGACATCTTGTCTA-----AWAAGCGCTATATAAAAACGAATTATTATTATT

**C**

Ther1 ACTAGCTGTGTGACCTTGGGCAAGTCACTTA-------ACCTCTCTGTGCCTCAGTTTCC-TCATCTGTAAAATGG---GGATAATAATAGCACCTACCTCA-CAGGGTTGTTG
Ther2 ACTAGCTGTGTGACCTTGGGCAAGTCACTTA-------ACCTCTCTGGGCCTCAGTTTCC-TCATCTGTAAAATGA---GGGGGTTGGACTAGATGGCCTCT-AAGGTCCCTT
Mon1 GTCTGCTGTGTGACCTTGGGCAAGTCACTTM-------ACTTCTCTGTGCCTCAGTTACC-TCATCTGTAAAATGG---GGATTAAGACTGTGAGCCCCACG-TGGGACAGGGA
Mar1 ACTAGCTGTGTGACCCTGGGCAAGTCACTTA-------AC-CCTGTTTGCCTCAGTTTCC-TCATCTGTAAAATGAGCTGGAGAAGGAAATGGCAAACCACTCCAGTATCTTTG
tuaMIRa ACTCACTGGGTGGCCTTGGGCAAGTCACTA-----------TCTCTCAGCCTCAGTT-CC-CCATCTGTAAAATGG---GAATAATAAT--GACCTACCTCA-CAGGGTTGTTG
tuaMIRb ACTCACTGGGTGGCCTTGGGCAAGTCACAA-----------CCTCTCAGCCTCAGTT-CC-CCATCTGTAAAATGG---GAATG
Coe2 ACTCACTGTGTGACCTTGGGCAAGTCACTTG-------ACCTCCTTGTGCCTCAGTTCCC-CCAGCTGTAAAATTG---GGTGGAAGCA-GGACCCCACTGA-AAATG
Squam3A GCTCACTGGGTGACCTTGGGCCAGTCACTG-----------TCTCTCAGCCTA---------------------------------------ACCTACCTCA-CAGGGTTGTTG
Squam3C GCCAGCTGGGTGACTTTGGGCCAGTCACTC-----------TCTCTCAGCCCA---------------------------------------ACCCACCTCA-CAGGGTTGTTG
tuaMIRc ACTCACTGGGTGACCTTGGGCCAGTCACAAT--------ATTCTCTCAGCCTA---------------------------------------ACCTACCTCA-CAGGGTTGTTG
Squam3B GCTAGCTGGGTGACCTTGGGCTAGTCACACTTCTTCTGARGTCTCTCAGCCCC---------------------------------------ACYCACCTCA-CAGRGTGTTTG
Mac1 ACTAGCTGTGTGACCTTGGGCAAGTCACTTA-------ACCCCAAT-TGCCTCATCCTGGGTCATCTCCAGTCATCCTGATGAATATCTGGTCACTGGATTCAGATGGCTCTGG
Mar3 ACTAGCTGTGTGACCCTGGGCAAGTCACTTA-------ACCCCAAT-TGCCTCA
WallSI4 ACTGGCTGTGTGACCCTGGGCAAGTCACTTA-------ACCTCTCAGTGCTCTAGGCAAC-TCTCTAAGACTATAA---GTTGCAGAGAAGGTGCTAACCTGCATTGGTAGAGG
Xt1 CAACTCCTTGTGACCCTGGACAAGTCACTTA-------ATCTCCTGGTGTCCCAGCATAC-TTAGTGCACCTATAA
