# Supplementary material for: New Ther1-derived SINE Squam3 in scaled reptiles
Source: Mob DNA. 2021 Mar 22;12:10. doi: 10.1186/s13100-021-00238-y (PMC7983390; doi:10.1186/s13100-021-00238-y)
Supplement: Supplementary file 3 — Additional file 3: Fig. S3. Alignment of LINE-derived regions of tuaMIRb and Ther1 and 3′-terminal sequences of several L2 LINEs. The origin and total length is given in parentheses. [file 13100_2021_238_MOESM3_ESM.pdf]

```
tuaMIRb                           GTGTSTGTCTGTCCCTGTGTTCCCCTCACCCTTGTCCATCTTAGATTGTAAGCCCACT---GGGCAGGGACC-TGTCTCAA------AAATCTGTGACTGTAAAGYGC-TRGTACA--------------CCTAG-----------TA-GCRCTATAGAAATGATTAGTAGTAGTAG
Ther1                                                                                                                              TGTAAAGCGCTTAGAACAGTG----CCTGGCACATAG-----------TAAGCGCTATATAAATGTTAGCTATTATTA-
L2_Mars (Opossum 3452bp)          GTATATATTTGTTTGCATGTTGTCT---------CCCCCATTAGATTGTAAGCTCCTT-GAGGGCAGGGACTGTCTTTTGC------CTCTTTTTGTATCCCCAGCGCTTAGCACAGTG----CCTGGCACATAG-----------TAGGCGCTTAATAAATGTTTATTGATTGATT
L2_Plat1e (Platypus 4160bp)       ATTTATTGCTATTGTTCTTGTCTGT-CCGTCTC-CCCCGATTAGACTGTAAGCCCGTCAAAGGGCAGGGACTGTCTCTATCTGTTACCGATTTGTACATTCCAAGCGCTTAGTACAGTG----CTCTGCACATAG-----------TAAGCGCTCAATAAATACTATTGAATGAATG
L2_Plat1r (Platypus 2361bp)       TCCTATCTGTAANTTATTTTAGTGT---CTGTCTCCCCCGCTAGATTGTAAGCTCCTT-GAGGGCAGGGATCATGTCTACTAACT--CTATTGTACTCTCCCAAGCGCTTAGTACAGTG----CTCTGCACACAG-----------TAGGCGCTCAATAAATACTATTGATTGATTG
L2_Plat1s (Platypus 2350bp)       TCCTATCTGTAAATTATTTT-GTGT---CTGTCTCCCCCGTTAGATTGTAAGCTCCTT-GAGGGCAGGGATCGTGTCTTCTACCT--CTATTGTACTCTCCCAAGCGCTTAGTACAGTG----CTCTGCACACAG-----------TAGGCGCTCAATAAATACTATTGATTGATTG
L2_Plat1t (Platypus 2377bp)       CACTATTTGTAAATAATTTTTGTCT---GTCTC-CCCCCATTAGATTGTAAGCTCCTT-GAGGGCAGGAA-CGTGTGTCTTGCTT--CTGTTGTACTCTCCCAAGCGCTTAGTACAGTG----CTTTGCACTCAG-----------TAGGCGCTCAATAAATACCATTGATTGATTG
L2-1_CPB (Turtle 3503bp)          GTCTGTCTGTATCCATCTGTTGTCT---CTTGTCTTATACTTAGATTGTAAGCTCTTT--GGGGCAGGGACCGTCTTTT---------TGTTCTGTGTTTGTACAGCGCCTAGCACAATGGGGTCCTGGTCCATGACTAGGGCTCCTAGGCGCTACGGTAATACAAATAATAATAAT
L2-5_CPB (Turtle 334bp)           GTTACTCTCACTCATTGCATCACGT---------CTAAAATTAGATTGTAAACTCTTC--AGGGCAGGGACCATGTCTT---------TTTATTTGTTCTGTAAAGCGCCTAGCACACTTT--------------------------TGGGTGCTGTAAAAATAATAATAATAA
```
