# Supplementary material for: New Ther1-derived SINE Squam3 in scaled reptiles
Source: Mob DNA. 2021 Mar 22;12:10. doi: 10.1186/s13100-021-00238-y (PMC7983390; doi:10.1186/s13100-021-00238-y)
Supplement: Supplementary file 4 — Additional file 4: Table S1. Squam3 copies found in individual NCBI sequences of squamate species not listed in Table 1. [file 13100_2021_238_MOESM4_ESM.docx]

|  | | | Family | Number of NCBI  sequences | Total hits | Squam3 subfamilies |
| --- | --- | --- | --- | --- | --- | --- |
| Gekkota | Gekkomorpha | | Eublepharidae | 1,677 | 440 | Squam3A (99%) |
|  |  |  |  |  |  | Squam3B (1%) |
|  |  |  | Gekkonidae | 32,944 | 10 | Squam3A (20%) |
|  |  |  |  |  |  | Squam3B (80%) |
|  |  |  | Phyllodactylidae | 12,364 | 0 |  |
|  |  |  | Sphaerodactylidae | 6,037 | 0 |  |
|  | Pygopodomorpha | | | 7,535 | 1 | Squam3A |
| Anguimorpha | | | | 6,712 | 1 | Squam3A |
| Iguania | | “Agamas” | Acrodonta | 24,499 | 0 |  |
|  |  | “Iguanas” | Dactyloidae | 22,026 | 2 | Squam3A |
|  |  |  | Iguanidae | 24,178 | 1 | Squam3A |
|  |  |  | Leiosauridae | 1,442 | 0 |  |
|  |  |  | Phrynosomatidae | 82,83 | 0 |  |
|  |  |  | Polychrotidae | 659 | 0 |  |
| Serpentes | | Caenophidia | Acrochordoidea | 279 | 0 |  |
|  |  |  | Colubridae (-6 genomes) | 35,124 | 4 | Squam3C |
|  |  |  | Dipsadidae | 17,248 | 2 | Squam3C |
|  |  |  | Elapidae (-11 genomes) | 490,627 | 134 | Squam3C |
|  |  |  | Elapidae *(Micrurus lemniscatus)* | 366,335 | 134 | Squam3A (10%) |
|  |  |  |  |  |  | Squam3C (90%) |
|  |  |  | Homalopsidae | 605 | 0 |  |
|  |  |  | Hydrophiidae | 2,052 | 0 |  |
|  |  |  | Lamprophiidae | 120,991 | 29 | Squam3A (3%) |
|  |  |  |  |  |  | Squam3C (97%) |
|  |  |  | Pareatidae | 813 | 0 |  |
|  |  |  | Viperidae | 73,9 | 27 | Squam3A (19%) |
|  |  |  |  |  |  | Squam3C (81%) |
|  |  |  | Xenodermatidae | 466 | 0 |  |
|  |  | Henophidia | | 6,251 | 1 | Squam3A |
|  |  | Scolecophidia | Typhlopoidea | 4,428 | 0 |  |
| Amphisbaenia | | | | 2,142 | 0 |  |
| Scincomorpha | | | Lacertidae | 32,629 | 27,413 | Squam3A (49%) |
|  |  |  |  |  |  | Squam3B (51%) |
|  |  |  | Teiioidea | 3,554 | 69 | Squam3A (93%) |
|  |  |  |  |  |  | Squam3B (6%) |
|  |  |  |  |  |  | Squam3С (1%) |
|  |  |  | other Scincomorpha | 60,445 | 3 | Squam3A |
| Dibamia | | | Dibamidae | 221 | 0 |  |
