# Supplementary material for: New Ther1-derived SINE Squam3 in scaled reptiles
Source: Mob DNA. 2021 Mar 22;12:10. doi: 10.1186/s13100-021-00238-y (PMC7983390; doi:10.1186/s13100-021-00238-y)
Supplement: Supplementary file 5 — Additional file 5: Table S2. Distribution of tuaMIR subfamilies in genomes of animals studied. [file 13100_2021_238_MOESM5_ESM.docx]

|  |  | **Family** | **Species** | **tuaMIRa** | **tuaMIRb** | **tuaMIRc** |
| --- | --- | --- | --- | --- | --- | --- |
| **Squamata** | Gekkota | Gekkonidae | *Gekko japonicus* | 285 | ─ | ─ |
|  |  |  | *Paroedura picta* | 17 | ─ | ─ |
|  |  | Eublepharidae | *Eublepharis macularius* | 142 | ─ | ─ |
|  | Lacertoidea | Lacertidae | *Darevskia valentini* | 9 | ─ | ─ |
|  |  |  | *Lacerta agilis* | 95 | ─ | ─ |
|  |  |  | *Lacerta bilineata* | 83 | ─ | ─ |
|  |  |  | *Lacerta viridis* | 71 | ─ | ─ |
|  |  |  | *Podarcis muralis* | 78 | ─ | ─ |
|  |  |  | *Zootica vivipara* | 57 | ─ | ─ |
|  |  | Teiidae | *Salvator merianae* | 2 | ─ | ─ |
|  | Serpentes | Colubridae | *Pantherophis guttatus* | 1 | ─ | ─ |
|  |  |  | *Pantherophis obsoletus* | 1 | ─ | ─ |
|  |  |  | *Ptyas mucosa* | 1 | ─ | ─ |
|  |  |  | *Thamnophis elegans* | 1 | ─ | ─ |
|  |  |  | *Thamnophis sirtalis* | 1 | ─ | ─ |
|  |  |  | *Thermophis baileyi* | 1 | ─ | ─ |
|  |  | Elapidae | *Emydocephalus ijimae* | 1 | ─ | ─ |
|  |  |  | *Hydrophis cyanocinctus* | 1 | ─ | ─ |
|  |  |  | *Hydrophis hardwickii* | 1 | ─ | ─ |
|  |  |  | *Hydrophis melanocephalus* | 1 | ─ | ─ |
|  |  |  | *Laticauda colubrina* | 1 | ─ | ─ |
|  |  |  | *Laticauda laticaudata* | 1 | ─ | ─ |
|  |  |  | *Naja naja* | 1 | ─ | ─ |
|  |  |  | *Notechis scutatus* | 1 | ─ | ─ |
|  |  |  | *Ophiophagus hannah* | 1 | ─ | ─ |
|  |  |  | *Pseudonaja textilis* | 1 | ─ | ─ |
|  |  | Pythonidae | *Python bivittatus* | 1 | ─ | ─ |
|  |  | Viperidae | *Crotalus horridus* | 1 | ─ | ─ |
|  |  |  | *Crotalus pyrrhus* | 1 | ─ | ─ |
|  |  |  | *Crotalus viridis viridis* | 1 | ─ | ─ |
|  |  |  | *Protobothrops flavoviridis* | 1 | ─ | ─ |
|  |  |  | *Protobothrops mucrosquamatus* | 1 | ─ | ─ |
|  |  |  | *Vipera berus berus* | 1 | ─ | ─ |
|  | Shinisauria | Shinisauridae | *Shinisaurus crocodilurus* | 507 | ─ | ─ |
|  | Anguimorpha | Anguidae | *Dopasia gracilis* | 61 | ─ | ─ |
|  | Varanoidea | Varanidae | *Varanus komodoensis* | 23 | ─ | ─ |
|  | Iguania | Agamidae | *Pogona vitticeps* | 2 | ─ | ─ |
|  |  | Dactyloidae | *Anolis carolinensis* | 17 | ─ | ─ |
| **Rhynchocephalia** | | | *Sphenodon punctatus* | 12983 | 12241 | 11871 |
| **Testudines** | | | *Trachemys scripta elegans* | 1362 | ─ | ─ |
| **Crocodilia** | | | *Crocodylus porosus* | 11 | ─ | ─ |
| **Aves** | | | *Gallus gallus* | 4 | ─ | ─ |
